# Chinese adult brain atlas with functional and white matter parcellation

**DOI:** 10.1038/s41597-022-01476-2

**Published:** 2022-06-20

**Authors:** Jingwen Zhu, Anqi Qiu

**Affiliations:** 1 Department of Biomedical Engineering, National University of Singapore, Singapore, Singapore; 2 The N.1 Institute for Health, National University of Singapore, Singapore, Singapore; 3 NUS (Suzhou) Research Institute, National University of Singapore, Suzhou, China; 4 School of Computer Engineering and Science, Shanghai University, Shanghai, China; 5 Institute of Data Science, National University of Singapore, Singapore, Singapore; 6 Department of Biomedical Engineering, The Johns Hopkins University, Baltimore, USA

**Keywords:** Brain, Brain

## Abstract

Brain atlases play important roles in studying anatomy and function of the brain. As increasing interests in multi-modal magnetic resonance imaging (MRI) approaches, such as combining structural MRI, diffusion weighted imaging (DWI), and resting-state functional MRI (rs-fMRI), there is a need to construct integrated brain atlases based on these three imaging modalities. This study constructed a multi-modal brain atlas for a Chinese aging population (n = 180, age: 22–79 years), which consists of a T1 atlas showing the brain morphology, a high angular resolution diffusion imaging (HARDI) atlas delineating the complex fiber architecture, and a rs-fMRI atlas reflecting brain intrinsic functional organization in one stereotaxic coordinate. We employed large deformation diffeomorphic metric mapping (LDDMM) and unbiased diffeomorphic atlas generation to simultaneously generate the T1 and HARDI atlases. Using spectral clustering, we generated 20 brain functional networks from rs-fMRI data. We demonstrated the use of the atlas to explore the coherent markers among the brain morphology, functional networks, and white matter tracts for aging and gender using joint independent component analysis.

## Abstract (summary)

**Table:** 

Measurement(s)	water content • water diffusion • blood oxygenation level-dependent signal
Technology Type(s)	Magnetic Resonance Imaging • Diffusion Tensor Imaging • Resting State Functional Connectivity Magnetic Resonance Imaging

## Background & Summary

Brain atlases have received a great deal of attention^1–3^, since they play important roles in studying anatomy and function of the brain in large populations^4–6^. Recently, there is an increasing interest in using multi-modal magnetic resonance imaging (MRI) approaches, such as structural MRI, diffusion weighted imaging (DWI), and resting-state functional MRI (rs-fMRI), for understanding brain development, degeneration, and abnormalities of gray matter and white matter tracts as well as functional organization. There is a need to construct integrated brain atlases based on structural MRI, DWI, and rs-fMRI such that the convoluted cortex, subcortical structures, white matter tracts, and functional networks are well aligned in a common stereotaxic coordinate space.

The well-known brain atlas, such as the MNI^3^ and ICBM^7^ atlases, was constructed based on structural T1-weighted images of Caucasian populations. Evidence has shown population differences in brain morphology among various ethnic groups^8–10^. In the past few years, several brain atlases targeting the Chinese population became available. Xing *et al*. constructed a set of brain structural atlases for various age and gender groups using 1000 Chinse adults^11^. The brain of Chinese is rounder in shape^8^, shorter in length and height but has a larger width to length ratio^12^ than that of Caucasians. Liang *et al*. utilized the population-matched brain structural atlas and achieved better segmentation performance for Chinese subjects than using those Caucasians-based brain structural atlases^12^. Yang *et al*. constructed a brain surface atlas based on a Chinese population and demonstrated better alignment and higher accuracy when registering a Chinese brain to the Chinese brain atlas compared to the brain atlas derived from a Caucasian population^13^. Functionally, Zhang *et al*., found that the language-related brain region was more strongly connected with the motor area and frontal region in Chinese compared to Caucasians^9^. Thus, there is a need to construct ethnic-specific brain structural and functional atlases.

Diffusion-weighted imaging (DWI) has been widely explored to understand the microstructure of the brain white matter based on the diffusion property of water molecules^14^. Diffusion tensor imaging (DTI) is the well-used model to quantify water diffusion^15^. Mori *et al*. constructed a DTI white matter atlas where projection and association white matter fibers are characterized in stereotaxic coordinates^16^. However, since DTI describes the axonal orientation of each voxel by a three-dimensional ellipsoid tensor, it has limited capability to resolve the complex architecture of crossing fibers^17^. To address the issue of multiple intravoxel fiber orientations, more complex diffusion imaging teachniques, such as high angular resolution diffusion imaging (HARDI)^18^, diffusion spectrum imaging (DSI)^19^ and q-ball imaging (QBI)^20^, have been developed to recover complex fiber architecture via an orientation distribution function (ODF), where the ODF is the angular profile of the diffusion probability density function of water molecules that characterizes white matter fiber orientations. Bloy *et al*. constructed the HARDI atlas for adolescents and employed an automated clustering algorithm to parcellate the white matter into regions with higher homogeneity of white matter fibers than those derived from conventional DTI^21^. Nevertheless, up to date, a Chinese-population-based brain structural atlas capable of modeling complex intravoxel fiber orientations is still missing. Also, there is a lack of brain atlases that integrate comprehensive white matter fibers and functional organization.

This study aimed to construct a multi-modal brain atlas for a Chinese aging population (age: 22 to 79 years), which consists of a structural T1 atlas showing the brain morphology, a HARDI atlas delineating the complex fiber architecture, and a rs-fMRI atlas reflecting brain intrinsic functional organization. We employed large deformation diffeomorphic metric mapping (LDDMM)^22^ and unbiased diffeomorphic atlas generation^23–25^ to simultaneously generate the structural T1 and HARDI atlases. Using spectral clustering, we generated 20 brain functional networks from rs-fMRI data. Further, we demonstrated the use of the atlas to explore the coherent markers among the brain morphology, functional networks, and white matter tracts for aging and gender using joint independent component analysis (ICA).

## Methods

### Subjects

This study was approved by the National University of Singapore Institutional Review Board and all participants provided written informed consent prior to participation.

**Fig. 1:**
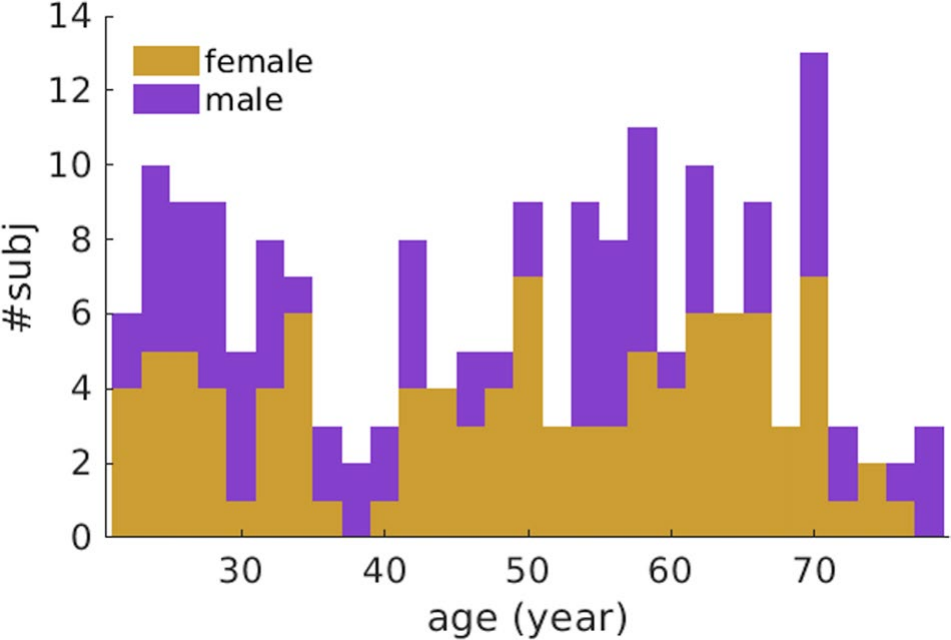
Age and sex distribution of subjects in this study.

Two hundred and fourteen healthy Chinese subjects aged 22 to 79 years old were recruited and screened for this study^26–29^. Chinese ethnicity was defined when both parents and grandparents are Chinese. Subjects with the following conditions were excluded: (1) major illnesses/surgery (heart, brain, kidney, lung surgery); (2) neurological or psychiatric disorders; (3) learning disability or attention deficit; (4) head injury with loss of consciousness; (5) non-removable metal objects on/in the body such as cardiac pacemaker; (6) diabetes or obesity; (7) Mini-Mental State Examination (MMSE) score less than 24. Additionally, this study included subjects with three brain image modalities, including T1-weighted MRI, resting-state fMRI (rs-fMRI), and high angular resolution diffusion image (HARDI) with small head motion^30^. As a result, this study included 180 subjects from 22 to 79 years old (77 males, 103 females). Figure 1 illustrates the age and sex distribution of subjects included in this study.

### MRI Acquisition and preprocessing

All subjects were scanned using a 3 T Siemens Magnetom Trio Tim scanner with a 32-channel head coil at the Clinical Imaging Research Centre of the National University of Singapore. The image protocols included: (i) high-resolution isotropic T_1_-weighted Magnetization Prepared Rapid Gradient Recalled Echo (MPRAGE; 192 slices, 1 mm thickness, sagittal acquisition, field of view 256 × 256 mm^2^, matrix = 256 × 256, repetition time = 2300 ms, echo time = 1.90 ms, inversion time = 900 ms, flip angle = 9°); (ii) isotropic axial resting-state functional MRI (rs-fMRI) imaging protocol (single-shot echo-planar imaging; 48 slices with 3 mm slice thickness, no inter-slice gaps, matrix = 64 × 64, field of view = 192 × 192 mm^2^, repetition time = 2300 ms, echo time = 25 ms, flip angle = 90°, scanning time = 8 min); (iii) isotropic high angular resolution diffusion imaging (HARDI; 48 slices of 3 mm thickness, with no inter-slice gaps, matrix: 96 × 96, field of view: 256 × 256 mm^2^, repetition time: 6800 ms, echo time: 85 ms, flip angle: 90°, 91 diffusion weighted images (DWIs) with b = 1150 s/mm^2^, 11 baseline images without diffusion weighting); (iv) isotropic T_2_-weighted imaging protocol (spin echo sequence; 48 slices with 3 mm slice thickness, no inter-slice gaps, matrix: 96 × 96, field of view: 256 × 256 mm^2^, repetition time: 2600 ms, echo time: 99 ms, flip angle: 150°). During the rs-fMRI scan, the subjects were asked to close their eyes.

The T_1_-weighted images were corrected for intensity inhomogeneity and were then skull-stripped using FreeSurfer (version 5.3.0)^31^. A post-processing quality check was conducted by one well-trained researcher based on the instruction given at https://surfer.nmr.mgh.harvard.edu/fswiki/FsTutorial/TroubleshootingData.

**Fig. 2:**
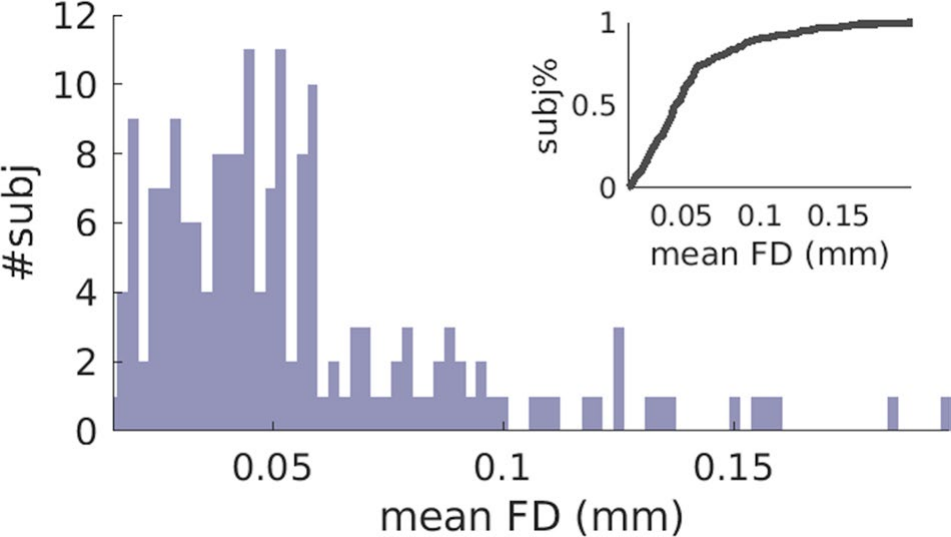
Mean framewise displacement (FD) distribution. Mean FD were smaller than 0.2 mm for all subjects, among which 90% had mean FD smaller than 0.1 mm.

The rs-fMRI data were preprocessed with slice timing, motion correction, skull stripping, band-pass filtering (0.01–0.08 Hz) and grand mean scaling of the data (to whole brain modal value of 100). Framewise displacement (head motion characteristics) was computed, and subjects with rs-fMRI data of framewise displacement (FD) greater than 0.5 mm were excluded from this study^30^. Figure 2 shows the mean framewise displacement distribution of rs-fMRI data among subjects. All the subjects in this study had the mean FD smaller than 0.2 mm. Among them, 90% had the mean FD smaller than 0.1 mm. Hence, head motion is regressed out from rs-fMRI using six parameters, and subsequently, this study regressed out CSF and white matter signals from rs-fMRI signal. Temporal band-pass filtering (0.01–0.08 *Hz*) was applied.

DWIs of each subject were first corrected for motion and eddy current distortions using mutual information for searching affine transformation to the image without diffusion weighting in FSL^32^. Within-subject, we followed the procedure detailed in Huang *et al*.^33^ to correct geometric distortion of the DWIs due to b_0_-susceptibility differences over the brain. Briefly, the T_2_-weighted image was considered as the anatomical reference. The deformation that carried the baseline image without diffusion weighting to the T_2_-weighted image characterized the geometric distortion of the DWI. For this, intra-subject registration was first performed using FLIRT^32,34^ to remove linear transformation (rotation and translation) between the diffusion weighted images and T_2_-weighted image. Then, large deformation diffeomorphic metric mapping (LDDMM)^22^ sought the optimal nonlinear transformation that deformed the baseline image without the diffusion weighting to the T_2_-weighted image. This diffeomorphic transformation was then applied to every diffusion weighted image in order to correct the nonlinear geometric distortion. The diffusion gradients were reoriented using the method proposed in Dhollander *et al*.^35^. Finally, we estimated the orientation distribution functions (ODFs) using the approach considering the solid angle constraint based on HARDI proposed in Aganj *et al*.^36^. The ODF is the angular profile of the diffusion probability density function of water molecules that characterizes white matter fiber orientations.

### Structural atlas generation using t1-weighted and diffusion weighted images

This study employed large deformation diffeomorphic metric mapping (LDDMM)^22,37^ to simultaneously generate multi-modal structural brain atlases, including structural T_1_-weighted MRI and HARDI. We adopted the unbiased diffeomorphic atlas generation procedure given in literature^23–25^. In particular, we formulated this atlas generation as

1 $$J\left({v}_{i,t},{I}_{atlas}{,\Psi }_{atlas}\right)=\mathop{\min }\limits_{{v}_{i,t}=\frac{\partial {\phi }_{i,t}}{\partial t}}{\sum }_{i=1}^{n}{\int }_{0}^{1}| | {v}_{i,t}| {| }_{V}^{2}\;dt+| | {I}_{atlas}\cdot {\phi }_{i,1}-{I}_{i}| {| }^{2}+| {| \log }_{{\Psi }_{atlas}\cdot {\phi }_{i,1}}{\Psi }_{i}| {| }_{{\Psi }_{atlas\cdot {\phi }_{i,1}}}^{2},$$

where *I*_*atlas*_, Ψ_*atlas*_ denote the structural T_1_-weighted atlas and the HARDI atlas represented by ODFs. *ϕ*_*i,t*_ and *v*_*i,t*_ are diffeomorphic transformation and its velocity at time, *t*, that transform the estimated atlas to the *i*^*th*^ subject space^22^. At *t* = 1, *ϕ*_*i*, 1_ transforms the T_1_-weighted and ODF atlases to the T_1_-weighted and ODF images of the *i*^*th*^ subject, respectively. $| | {v}_{i,t}| {| }_{V}^{2}$ is the regularization term to constraint the smoothness of the velocity field in a Hilbert space, *V*. ||*I*_*atlas*_∙*ϕ*_i, 1_-*I*_*i*_||^2^ quantifies the intensity difference between the transformed T_1_-weighted atlas and the T_1_-weighted image of the *i*^*th*^ subject. $| {| \log {}_{{\Psi }_{atlas}\cdot {\phi }_{i,1}}\Psi }_{i}| {| }_{{\Psi }_{atlas}\cdot {\phi }_{i,1}}^{2}$ is defined as Fisher-Rao metric in the statistical manifold of ODFs. The mathematical definition of this Fisher-Rao metric was detailed in Du *et al*.^25^. It quantifies the angle between the transformed atlas ODF, Ψ_*atlas*_∙*ϕ*_*i*, 1_, and the *i*^*th*^ subject ODF, Ψ_*i*_.

To solve Eq. (1), single-subject structural and HARDI ODF images were used as an initial atlas. Structural MRI and HARDI ODF of the initial atlas were simultaneously aligned to those of individual subjects via LDDMM transformations^22,37^. The structural T_1_-weighted atlas was obtained by averaging the deformed images of individuals. The HARDI atlas was computed by averaging the deformed ODF of individuals based on mean of ODFs in the ODF statistical manifold^25^. We repeated this process for three iterations to obtain the final structural T_1_-weighted image and HARDI ODF atlases as the intensity change of the atlases obtained from the second and third iterations was less than 5%. Last, we mapped manually labelled white matter parcels from the JHU-MNI-SS atlas^38^ into the HARDI atlas via diffeomorphic transformation obtained using LDDMM, resulting in 94 deep white matter parcels (see the annotation in Supplementary Table S1).

In this study, we employed ODF to represent the direction of white matter fibers and scalar image, generalized fractional anisotropy (GFA), to quantify how the shape of ODF is deviated from a unit sphere.

### Functional atlas parcellation

In this study, the functional parcellation for the cortical and subcortical regions was constructed based on the rs-fMRI data of all subjects in the above structural atlas space. Individual rs-fMRI data were aligned to our structural T_1_-weighted atlas created above via LDDMM between the respective T_1_-weighted images. Given that spectral approaches are robust well-proven methods for parcellating the brain, as they are especially suitable for solving general problems, and tend to provide partitions with more balanced sizes compared to other clustering methods, such as hierarchical clustering^39,40^, we employed spectral clustering to construct the functional parcellation from rs-fMRI data^41^. First, the gray matter mask was constructed by subtracting the white matter mask from the structural atlas, where the white matter mask contained 94 white matter parcels. Second, a voxel-pairwise similarity matrix was computed via Pearson’s correlation between the time course of any two voxels in the gray matter mask for individual subjects. Negative functional connectivities were retained and proceeded with the same computational procedure as for positive connectivities. A group similarity matrix was averaged across individual functional connectivity matrices and was then standardized to be maximum of one and minimum of zero. Third, spectral clustering was applied on the group average similarity matrix. Silhouette index that indicates the balance between intra-cluster compactness and inter-cluster separation was used to determine the number of clusters^42^. A higher value of the Silhouette index indicates a better clustering result. We shall call a functional cluster as a functional network in the rest of the paper.

To evaluate the reproducibility of the functional parcellation, we repeated the spectral clustering analysis via leave-one-out cross-validation. We then computed the overlap ratios between the new and original functional networks for each repetition^43^. Moreover, we compared our parcellation with that generated by hierarchical clustering using Ward’s algorithm^44^. Our study chose Wald’s hierarchical clustering method since it is a well-proved hierarchical clustering algorithm in terms of its robustness to generate the functional parcellation. Previous studies showed that Wald’s method is superior to several brain functional parcellation methods, such as geometric clustering and k-means clustering^45^. Our study computed the overlap ratio between our parcellation and that generated from Wald’s hierarchical clustering method.

## Data Records

### Structural MRI and hardi brain atlases

**Fig. 3:**
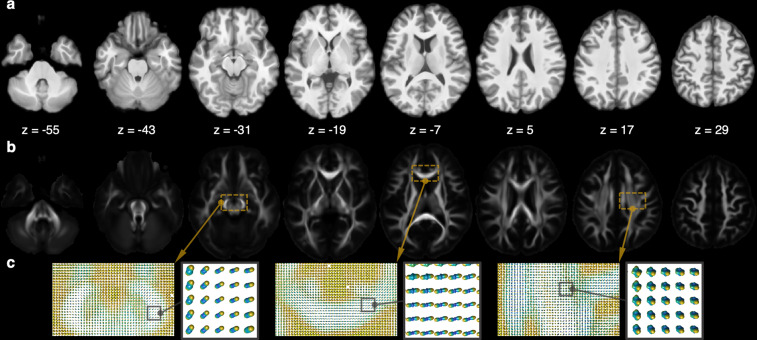
Structural atlas. Panels (**a**,**b**) respectively illustrate the structural T_1_-weighted atlas and HARDI GFA atlas. Panel (**c**) enlarges the white matter region in the dashed box and illustrates the ODF representing the orientation of white matter fibers.

Figure 3 illustrates the structural T_1_-weighted atlas (panel a) and HARDI GFA (panel b). The GFA, similar to FA, characterizes the overall shape of the white matter fiber distribution relative to a unit sphere. The larger GFA value (1 as max value) indicates more complex fiber orientation, while GFA of zero indicates that the ODF is in a spherical shape. Figure 3c shows the ODF in the midbrain, corpus callosum, superior longitudinal fasciculus (SLF). The first two show synchronized fiber orientation and the last one shows cross fibers in the SLF.

### Resting-state functional brain atlas

**Fig. 4:**
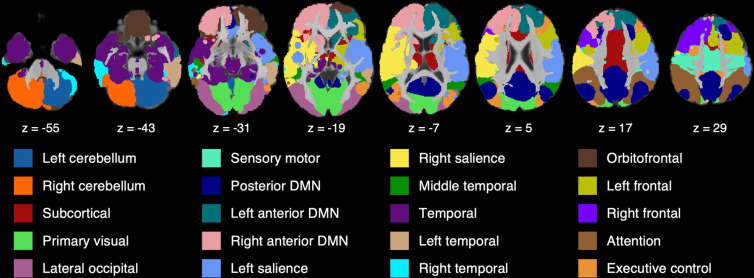
Resting-state functional atlas. This atlas consists of 20 functional networks that are illustrated in the axial view of the brain.

**Fig. 5:**
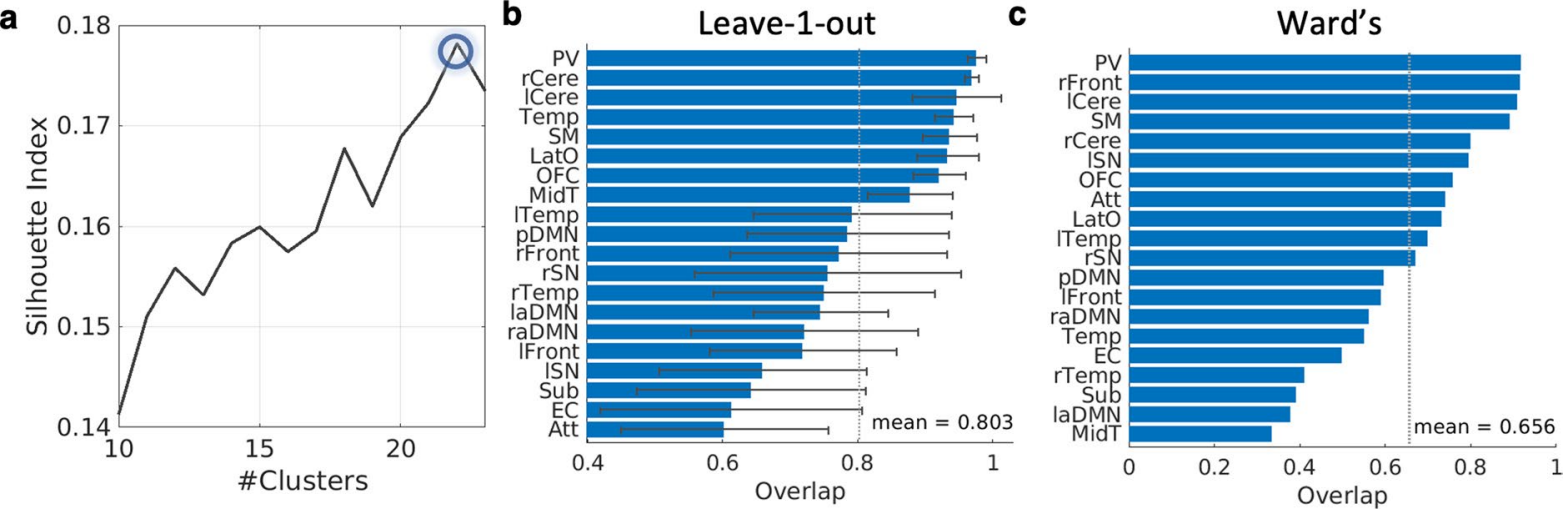
Model selection and reproducibility of brain functional parcellation. Silhouette index indicates that the gray matter region can be clustered into 22 functional networks (**a**). leave-one-out cross-validation results showed the average overlap ratio between the reproduced results and our 20 meaningful functional networks derived from the full dataset across 20 meaningful functional networks was 80.3% (**b**). The average overlap ratio between the parcellation results from Ward’s algorithm and 20 meaningful networks from our main analysis was 65.5% (**c**).

**Fig. 6:**
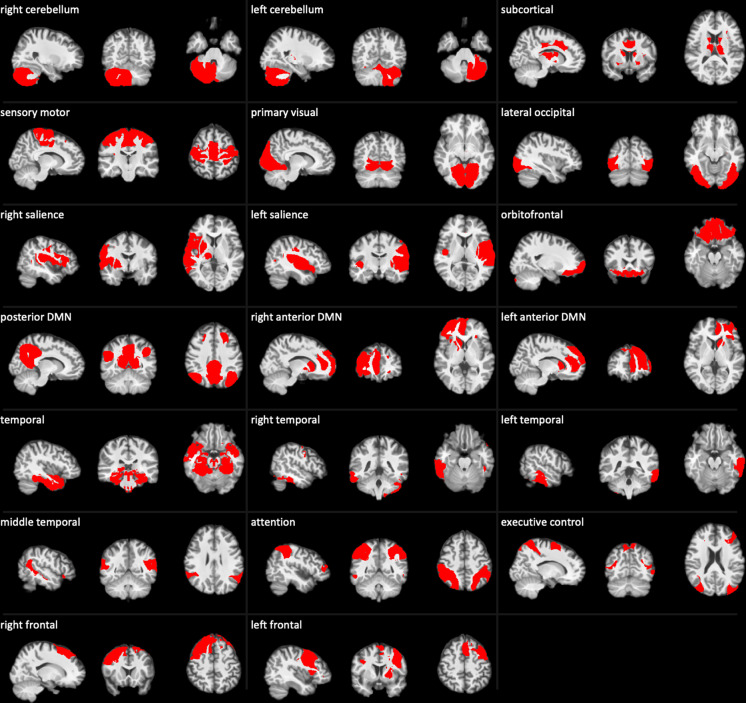
The 20 functional brain networks.

The spectral cluster algorithm identified 22 functional networks from the rs-fMRI data. Figure 5a illustrates Silhouette index, suggesting the maximal value of Silhouette index reached when the brain was classified into the 22 functional networks. Through the visual inspection, we discarded 2 functional networks that were mainly located at the white matter and cerebrospinal fluid (CSF) regions, resulting in the final 20 functional networks. Figure 4 illustrates the 20 functional networks in the axial view of the brain and Fig. 6 provides the three views of each network. Supplementary Table S2 lists the descriptive information of the 20 functional networks.

#### Cerebellar and subcortical networks

The cerebellum was partitioned into two functional networks, including the left and right cerebellum, while the subcortical network included the basal ganglia and thalamus as well as the middle cingulate. The first row in Fig. 6 illustrates the two cerebellar networks and one subcortical network.

#### Primary sensory networks

The 20 functional networks included three primary sensory networks, namely, the sensory motor network located at the motor cortex, the primary visual network located at cuneus, and the lateral occipital network (the second row in Fig. 6).

#### DMN and salience

The default mode region was fractionated into three functional networks (the fourth row in Fig. 6). The precuneus, post cingulate, and angular gyrus made up the posterior default mode network (DMN). The medial frontal, anterior cingulate, and caudate were further clustered into the left and right anterior DMNs.

Similarly, the insula, putamen, and thalamus were fractionated into two unilateral functional networks, namely, the left and right salience (the first two panels on the third row in Fig. 6).

#### Temporal networks

The temporal lobe was partitioned into four functional networks. The middle temporal network was located at the middle temporal region (the first panel on the sixth row in Fig. 6), and the temporal network covered the temporal pole, hippocampus, parahippocampus, and amygdala (the first panel on the fifth row in Fig. 6). Both networks displayed bilateral patterns. The inferior and middle temporal regions were further partitioned into two unilateral functional networks, namely, the left and the right temporal functional networks (the fifth row in Fig. 6).

#### Frontoparietal networks

The frontoparietal cortex was clustered into five functional networks. The orbitofrontal network clearly delineated the orbitofrontal cortex. The attention network was located at the inferior parietal cortex and displayed a bilateral pattern. The executive control network showed a dispersed bilateral pattern in the parietal, temporal, and occipital cortex, but diminished the left counterpart in the superior frontal cortex. The superior frontal cortex together with the anterior cingulate and basal ganglia was further clustered into the left and right frontal networks (the last row in Fig. 6).

### Integrated structural and functional atlas

**Fig. 7:**
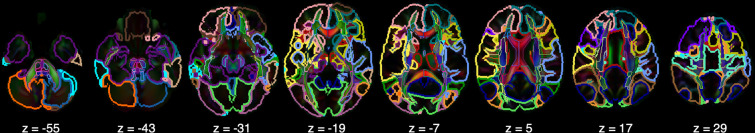
Integrated structural and functional brain atlas. The colored outline delineates the 20 functional networks, where the color scheme is consistent with those in Fig. 2. The color map in the white matter shows the white matter parcellation.

Figure 7 illustrates the integrated structural and functional atlas, where the gray matter was parcellated into 20 functional networks and the white matter was segmented into 94 white matter tracts. The atlas and all the image data used in this paper are available at NITRC https://www.nitrc.org/projects/adultatlas^46^.

## Technical Validation

**Fig. 8:**
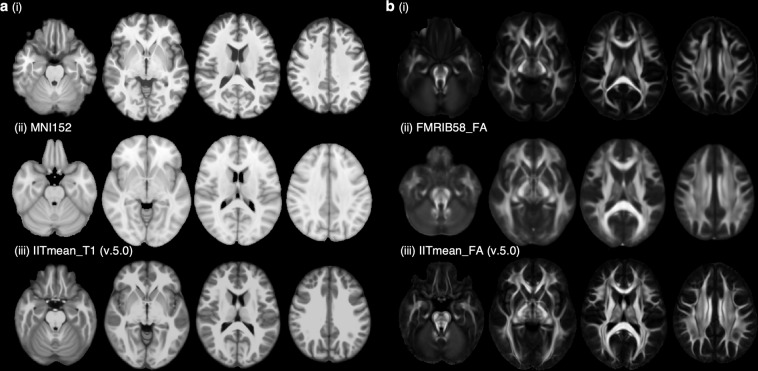
Comparison of structural atlases. Panel (**a**) illustrates the structural T_1_-weighted atlas in comparison with MNI152 T_1_-weighted atlas (ii) and IITmean T_1_-weighted atlas (iii). Panel (**b**) illustrates the HARDI GFA atlas in comparison with FMRIB58 FA atlas (ii) and IITmean FA atlas (iii).

Figure 8 demonstrates our structural atlases including the T_1_-weighted atlas (panel a) and the HARDI GFA atlas (panel b) in comparison with well-established adult atlases created based on different ethnic subjects. Visually, our T_1_-weighted atlas (panel a, (i)) offered better contrast than the MNI152 atlas (panel a, (ii)). The patterns of sulci and gyri in our T_1_-weighted atlas were largely agreed with those in the MNI152 atlas (panel a, (ii)) and those in the IIT T_1_ atlas^47^ (panel a, (iii)). On the other hand, the anatomical details of the white matter are clearer in our HARDI GFA atlas (panel b, (i)) than those in the FMRIB58 FA atlas (FMRIB, Oxford, UK) (panel b, (ii)). Major white matter tracts, including the corpus callosum, corona radiata, internal capsule, external capsule, superior and inferior longitudinal fasciculus, and small features, including the anterior commissure and superficial white matters, in the IIT FA atlas^48^ (panel b, (iii)) were observed in our HARDI GFA atlas as well (panel b, (i)).

Figure 5b shows the reproducibility of functional parcellation through leave-one-out cross-validation. Among the 20 functional networks derived from the full dataset, the average overlap ratio between the reproduced results and those from our main analysis was 80.3%. The cerebellum and primary sensory networks, including the primary visual, lateral occipital, and sensory motor, demonstrated high consistency among reproduced results with an average overlap ratio greater than 90%. On the other hand, the subcortical network and association networks, including the attention, executive control, salience networks, default mode networks, and temporal networks, were relatively variable compared to the primary sensory networks.

Figure 5c shows the overlap ratio of functional parcels using spectral clustering and those using Ward’s algorithm. The average overlap ratio between the parcellation scheme from Ward’s algorithm and that from our main analysis over all 20 meaningful functional networks was 65.5%. Similar to reproducibility results from leave-one-out cross-validation, the cerebellum and primary sensory networks from Ward’s algorithm were highly consistent with those derived from our main analysis, while higher-ordered functional networks from Ward’s algorithm, in general, showed a lower overlap ratio with those derived from our main analysis. Notably, three networks in the association cortex, namely, attention network and left and salience networks, were well reproduced via Ward’s algorithm.

## Usage Notes

We illustrated the use of the integrated structural and functional atlas to examine the synchronized pattern of the brain morphology, functional networks, and white matter tracts due to gender and age. For this, we applied joint independent component analysis (ICA) to incorporate the structure and functional measures. For functional data, we calculated the network-level functional connectivity matrix based on our 20 functional networks. For structural T1-weighted data, we employed cortical thickness to characterize the cortical ribbon. We mapped our age-appropriate functional atlas into cortical surfaces and calculated the cortical thickness averaged over each functional network. The average GFA value of each white matter tract was computed based on the HARDI atlas. As a result, the input matrix for multi-modal fusion consisted of 34 measures of thickness, 94 measures from the white matter parcels, and 210 functional connections, including 190 inter-network functional connections and 20 intra-network functional connections, from the functional atlas for each subject.

**Fig. 9:**
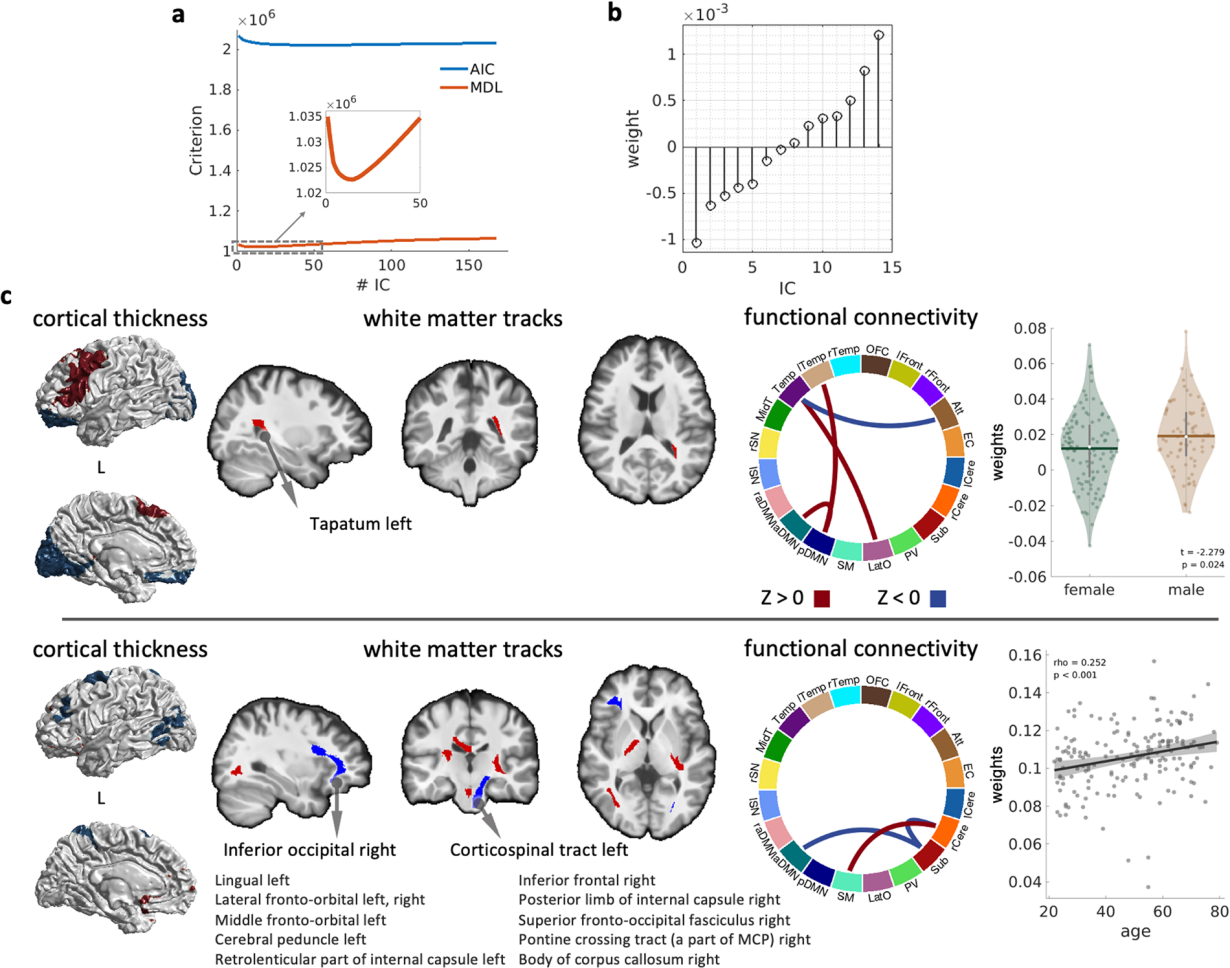
Joint independent component analysis on cortical thickness, functional network connectivity, and GFA of white matter tracts. Panel (**a**) shows the AIC and MDL in relation with the number of ICs. According to the average loading of those 14 ICs (**b**), we illustrated the IC with the largest negative loading and its association with gender (c, top panel), and the IC with the largest negative loading and its association with age (c, bottom panel).

We chose 14 IC components based on Akaike information criterion (AIC) and minimum description length (MDL) estimation^49^ (Fig. 9a). Figure 9b illustrates the loadings for each IC. We then investigated the age and gender differences of those components based on Pearson’s correlation and *t*-test, respectively. The top row in Fig. 9c illustrates the IC with the largest negative loading. The loading of this component did not show significant age-related changes but displayed significant gender difference. This gender-related component consisted of the cortical thickness in the left frontal, orbitofrontal, and primary visual networks, the white matter tract connecting to the left temporal lobe, and the functional connectivity between the temporal region and attention network, posterior DMN, and lateral occipital network. This component may reflect the different brain recruitment during language tasks between males and females^50^. The bottom row in Fig. 9c illustrates the IC with the largest positive loading that shows a significant age-related increase (scatter plot on the last panel). This age-related component consisted of the cortical thickness in the executive control network, functional connectivity between the right cerebellum and sensory motor and subcortical networks, between the subcortical and left anterior DMN, and dispersed white matter tracts connecting the subcortico-cerebellar region to the cerebral cortex. This component might reflect the age-related changes in the functional connectivity between the subcortical and cortical cortex.

## Supplementary information

Supplementary Table S1

Supplementary Table S2

## Data Availability

The atlas is available at and all the image data used in this paper are available at https://www.nitrc.org/projects/adultatlas. Code for the atlas generation can be found at https://github.com/bieqa/AdultBrainAtlas.
